# Self-awareness in Dementia: a Taxonomy of Processes, Overview of Findings, and Integrative Framework

**DOI:** 10.1007/s11910-021-01155-6

**Published:** 2021-11-24

**Authors:** Daniel C. Mograbi, Jonathan Huntley, Hugo Critchley

**Affiliations:** 1 Department of Psychology, Pontifical Catholic University of Rio de Janeiro, Rio de Janeiro, Brazil; 2 Institute of Psychiatry, Psychology & Neuroscience, King’s College London, De Crespigny Park, PO Box 078, London, SE5 8AF UK; 3 Division of Psychiatry, University College London, London, UK; 4 Wellcome Centre for Human Neuroimaging, University College London, London, UK; 5 Sackler Centre for Consciousness Science, University of Sussex, Brighton, UK; 6 Psychiatry Department of Neuroscience, Brighton and Sussex Medical School, Brighton, UK

**Keywords:** Self-awareness, Interoception, Agency, Metacognition, Selfhood, Dementia

## Abstract

**Purpose of Review:**

Self-awareness, the capacity of becoming the object of one’s own awareness, has been a frontier of knowledge, but only recently scientific approaches to the theme have advanced. Self-awareness has important clinical implications, and a finer understanding of this concept may improve the clinical management of people with dementia. The current article aims to explore self-awareness, from a neurobiological perspective, in dementia.

**Recent Findings:**

A taxonomy of self-awareness processes is presented, discussing how these can be structured across different levels of cognitive complexity. Findings on self-awareness in dementia are reviewed, indicating the relative preservation of capacities such as body ownership and agency, despite impairments in higher-level cognitive processes, such as autobiographical memory and emotional regulation.

**Summary:**

An integrative framework, based on predictive coding and compensatory abilities linked to the resilience of self-awareness in dementia, is discussed, highlighting possible avenues for future research into the topic.

## Introduction

Self-awareness is fundamental to psychology and the humanities, yet the topic remains at the frontier of scientific knowledge. Over centuries, distinct philosophical and religious traditions have grappled with the subject. In ancient Greece, the maxim ‘Know thyself’ greeted visitors seeking foresight from the oracle at the Temple of Apollo at Delphi. Eastern traditions similarly emphasise the importance of insight and self-knowledge [1]. However, only recently has the importance of self-awareness been established as a central concept for scientific investigation, and to which scientific methods can be applied to explore and characterise this phenomenon [2].

Self-awareness can be defined as the capacity of becoming the object of one’s own awareness [3]. This definition suggests a unitary, continuous model of self that can be observed and that itself acts as the observer. The self-model may be implicit but is revealed because it can enter conscious awareness, where it can be scrutinised. Nevertheless, a multiplicity of self-processes is encompassed within such a self-model [4, 5]. Self-awareness is thus dynamic, with inter-related yet heterogeneous aspects [6], each potentially dominating the self-model or awareness at a given time. For example, *bodily self-awareness*, including interoception (representation of one’s internal body state) and proprioception (representation of one’s body in space), can be phenomenologically distinguished from *metacognitive thinking* (awareness and evaluation of own thoughts), yet both are forms of self-awareness. Aspects of emotion, including the identification of affective feelings, may in part bridge these axes. Importantly, clinical conditions can lead to selective deficits of self-awareness, wherein specific abilities may be compromised, according to the profile of structural and functional brain alterations [7•, 8].

Self-awareness has important implications, notably from a clinical perspective. Recognising and understanding alterations in self-awareness within patient groups may enhance clinical management of neurological and psychiatric conditions and inform novel therapeutic interventions. For example, rehabilitation of neurological patients with impaired self-awareness might best employ implicit (non-aware) adaptations [9, 10], whereas therapies for specific psychiatric ‘disorders of self’, in conditions including psychosis and bipolar disorder, can respond to targeted metacognitive training [11, 12]. Additionally, a comprehensive understanding of the neurobiological mechanisms of self-awareness and its distinct components will shed light on fundamental principles of brain functioning. This can have profound implications that may lead to a reappraisal of extant models of general cognitive and emotional processes. A finer understanding of self-awareness may also have broader social relevance; for example, quantifying the extent to which individuals, patient groups, species, and devices are self-aware may reshape operational definitions of legal responsibility and culpability.

Exploring self-awareness is particularly relevant in the context of dementia. Neurodegenerative processes impact long-established expressions of identity to the detriment of self-management and interaction with others, including family and carers. Here, the recognition of self-awareness as a heterogeneous process is composed of different elements that may be differentially and variably affected and may guide a finer evaluation of preserved and impaired abilities in dementia. This recognition is crucial to avoid care practices expressing ‘malignant social psychology’ [13], in which ‘personhood’ is neglectfully devalued alongside the progressive and cognitively debilitating effects of dementia. Individual and institutional caregiving practices may have depersonalizing, disempowering elements that stigmatize people with dementia (PwD), and further add to disability [13]. Here, a greater appreciation of how aspects of self-awareness are compromised or retained in dementia may enhance person-centred care practices that foster preserved abilities and retain personhood.

Considering the above, the current article appraises self-awareness from a neurobiological perspective and discusses how evidence obtained from the study of PwD increases our understanding of how self-awareness is organised in the human brain. For this purpose, distinct self-awareness processes will be considered, followed by a discussion on how self-awareness can be structured across different levels of cognitive complexity. Data obtained with PwD will be discussed in relation to self-awareness, leading to the suggestion of an integrative framework helping to establish future empirical research into this topic.

## A Taxonomy of Self-awareness

As indicated above, self-awareness encompasses multiple dissociable processes (Table 1). This perspective is particularly relevant to understanding pathological neurocognitive alterations, for example in the case of dementia.

**Table 1:** A taxonomy of self-awareness

Self-awareness processes	Definition	Neural correlates
Interoception	Sense of the physiological conditions of the entire body	Insula, subcortical, and brain stem regions linked to homeostatic processes
Proprioception and body ownership	Mapping of the relative position of body parts, and the feeling of owning a body, respectively	Somatosensory cortex, thalamus, basal ganglia, cerebellum, and motor areas
Agency	Sense of generating our own actions	Angular gyrus and other temporoparietal regions, motor areas
Metacognition	Monitoring, knowledge, and regulation of cognition	Anterior cingulate and frontal cortical regions
Emotional regulation	Monitoring and regulation of emotion	Cortical-subcortical loops
Autobiographical memory	Records of self-information, including specific episodes and general knowledge about oneself	Medial and lateral temporal regions and medial prefrontal cortex

Theoretically, interoception, defined as the sense of physiological conditions of the body [14], is at the core of self-representation and thus fundamental to self-awareness. Life depends on maintaining stability in the internal physiological state of the body through low-level homeostatic reflexes, and via adaptive and anticipatory (allostatic) responses that are coordinated across organ systems and motivate adaptive behaviours. The information flow from the body to the brain is integrated to provide an inescapable dynamic representation of the physical self that coordinates behavioural responses to meet current and future needs. While much is achieved implicitly, through autonomic reflexes, awareness first emerges as bodily sensations and affective states. Thus, interoception and its organism-level control provide the basis to both a unitary biological self-object and the representation of this self as ‘an agent’ [15]. The latter is crystalized through interoceptive policies that adjust internal physiology in anticipatory and adaptive allostasis [15] and extend the biological self-model to encompass other correlated sources of sensory information [15, 16].

One influential model describes distinct psychological dimensions of interoception [17]. This model reflects different levels at which interoceptive information can be perceived and has proved useful in tightening nomenclature when studying contributions of interoception to cognitive and emotional processes. Within the brain, lower-level interoceptive inputs into the brainstem and subcortical regions support homeostatic reflexes, yet feed up into ‘viscerosensory cortex’ in the insula, where interoceptive information is integrated with other sensory and contextual information [18••]. Forward representation within anterior insular cortex supports feeling states through conscious access and appraisal of interoceptive sensations (putatively through comparison with expectations encoded as an efference copy of visceromotor drive from anterior cingulate cortex; [19]). In this way, generalised feelings concerning the overall state of internal bodily physiology can be evaluated against intended bodily arousal states appropriate for a selected behaviour.

Bodily self-awareness is bound by the representation of the physical extent of the body and the relative position of body parts in space, i.e. *proprioception*. Proprioception augments feelings of owning a body, and its self-location [20]. This outward focus of proprioception can also be differentiated from interoception (to which it is closely bound; [18••]). Within the brain, the somatosensory cortex, temporoparietal junctions, and a wider network of associated brain regions, including the thalamus, basal ganglia, cerebellum, and motor areas, contribute to the dynamic representation of proprioceptive information and awareness of one’s physical presence within a spatial context (for a meta-analysis, see [21]). Aberrant functioning of this system is implicated in disorders of body ownership, in cases such as somatoparaphrenia [22].

In contrast to the largely implicit nature of interoceptive information, there is greater conscious access to, and awareness of, proprioceptive information and its direct relationship to the control of bodily state and position. This enhances the feeling of owning a body. The sense of generating our own actions, termed agency, is a key component of self-awareness [23, 24]. Agency is closely tied to action, and the feeling of agency encompasses self-efficacy through the cognitive feeling of voluntary control of one’s actions. Unsurprisingly, models of agency typically suggest that an intention to move leads to a motor command, with a copy of that command (efference copy) being generated to predict the consequence of the command. This prediction is compared with feedback of the consequences of action, giving rise, when there is no mismatch, to the feeling of agency [23]. The parietal cortex, in particular temporo-parietal regions, notably the angular gyrus, is particularly implicated in agency [23, 25]. As suggested above, interoceptive mechanisms may enhance feelings of agency through the viscerosensory consequences of ‘central command’ and efference copy from motor and cingulate cortices into the insular cortex [19, 26]. The presence of Von Economo Neurons in the anterior insula and anterior cingulate cortices in certain ‘higher’ species, including great apes and especially humans, putatively links the evolution of specialised neural circuitry (cortical-brainstem systems potentially involved in interoceptive control) to greater self-awareness and insight [27, 28].

Metacognition, heuristically ‘thinking about thinking’, is fundamental to self-awareness, not least because it encompasses the appraisal of mental processes thoughts, emotional feelings, and perceptual representations. Here, the self is implicit; mental processes (cognitions) are objectified and ‘owned’ and appraised from a typically unitary self-perspective. Metacognitive measures of insight represent a strong index of awareness, particularly during psychological task performance: Both over- or under-estimating one’s performance (or ability) translates to poor metacognitive awareness (insight), compared to knowing accurately if one performs well or badly. More generally, metacognition is a higher cognitive form of self-awareness, operationalised as the monitoring, knowledge, and regulation of cognitions [29]. These cognitions may involve previous information, experiences, emotions, and goals, including the formulation of cognitive strategies e.g. prospective problem solving [29]. Within the brain, metacognition is primarily linked to the medial and dorsolateral frontal cortical function [30]. For example, electroencephalography studies indicate that the dorsal anterior cingulate is a crucial structure in error monitoring processes [31] and associated psychophysiological reactions [32]. The ventromedial prefrontal cortex is engaged in self-referential processing and, relatedly, adjacent orbitofrontal cortices represent relative rewards, and the emotions engendered, including whether a reward was less than expected [33]. Similarly, the dorsolateral prefrontal cortex is recruited during self-monitoring of task performance [34••]. Given that metacognition is primarily measured through self-report, the extent to which species other than humans can show metacognitive abilities is a source of debate. A few studies suggest metacognitive ability in primates and rodents (e.g. [35]), but methodological issues typically prevent firm conclusions [36].

Although generally distinguished from metacognition, emotional regulation engages similar processes, including thought and response monitoring and strategic regulation, but applied to emotion; i.e. valenced cognitions, affective feelings, and arousal. Emotional regulation strategies include changing the antecedents of an unwanted emotional experience, or regulating the emotional responses that form part of the experience [37]. Examples of ‘mitigative’ antecedent-focused emotional regulation include selective avoidance of evoking situations, attempts to modify such situations, attentional redeployment (e.g. distraction, mindfulness), and cognitive reappraisal [37]. Response-based regulation is generally less adaptive and includes strategies such as emotion suppression, substance abuse, and self-harm [38]. Antecedent-focused emotional regulation therefore entails a stronger model of self that can be protected into future counterfactual scenarios. The mechanisms linked to emotional regulation typically reflect interactions across cortical-subcortical neural substrates (e.g. amygdala and basal ganglia) [39]. For instance, prefrontal and anterior cingulate cortices are implicated in the active deployment of attention in emotional regulation (including distraction and reappraisal, and response suppression) [40].

Finally, in order to be available for future use, information about the self needs to be registered in time (as well as space). Self-based memories are referred to as autobiographical memory. Autobiographical memory can be divided into incident memory (encoding and recollection of specific episodes) and personal semantics (general knowledge about oneself; [41]). This conceptual distinction is supported by neuroimaging and lesion studies that distinguish brain networks for episodic and semantic components of autobiographical memories [41, 42]. Notably, the medial temporal cortex, including the hippocampus and surrounding cortical regions, is crucial for the encoding and recollection of incident memory [43]. Personal semantics share similar areas, but also engage regions linked to general semantic memory (e.g. lateral temporal lobe) and regions implicated in self-referential processing (e.g. ventromedial prefrontal cortex) [44]. Moreover, self-related autobiographical and spatial memories share similar medial temporal neural substrates (e.g. hippocampus). There are conceptual similarities between memory for events in time and the spatial mapping and navigation of locations, including ‘perspective-taking’. However, it is unclear how interdependent are the representations of self in time and place.

## Levels of Self-awareness

The facets of self-awareness highlighted above are associated with dissociable target components of self-representation and distinct expressions of awareness. These abilities vary in the degree to which they draw upon higher (integrative) cognitive processing. Nevertheless, such expressions of self-awareness are manifest across different levels of complexity [45]. In this sense, self-awareness is not as simply as a function of either lower- or higher-order processing, but rather as a set of capacities transcending representational levels.

For example, a research framework for conceptualising interoceptive abilities distinguishes between interoceptive accuracy, sensibility, and awareness [17]. Accuracy can be defined as the objective ability to detect internal states, interoceptive sensibility represents dispositional tendencies to be internally self-focused, while metacognitive interoceptive awareness (insight) indicates the level of self-knowledge about the degree to which one can accurately judge interoceptive sensations [17]. Interestingly, dissociative symptoms, such as depersonalization, representing a symptomatic partial disturbance of self-representation, are associated with deficits in metacognitive interoception [46]. In relation to agency, a distinction is proposed between the feeling of agency and the judgement of agency. The feeling of agency captures implicit non-conceptual feelings of being an agent of action. In contrast, the higher-level explicit judgement of agency is influenced by prior knowledge, expectations, and beliefs [24, 47].

Similarly, self-awareness manifest through metacognition is not exclusively a higher-order process, since it involves early ‘preconscious’ representations, apparent for example in electrophysiological signatures of error-monitoring, such as the early error-related negativity (ERN) and subsequent error positivity (Pe), which can be selectively compromised in neurological disorders [48]. Homologous mechanisms are shown to operate also in non-human primates [49], and, in humans, the Pe is associated with both awareness of error and accompanying psychophysiological responses [50]. Evidence from studies of children [51] and patients with neurodegenerative conditions [52, 53] also indicates that metacognitive processing can operate implicitly, impacting appraisals and decision-making of intermediate complexity. Metacognitive beliefs also enter into and self-appraisal of cognitive ability and performance at higher levels of complexity (e.g. [54]).

Even in the case of autobiographical memory, characterised conceptually as composed of episodic and semantic memories, varying levels of complexity in self-processing can be distinguished. For instance, implicit forms of memory, such as motor habits linked to procedural memory, embody important aspects of self-information that determine how to engage in actions and select between motor behaviours [55]. Potentially, low-level long-term representations, e.g. of a core ‘biological self’, not linked to autonoetic consciousness and re-experiencing, may be at the foundation of higher-order autobiographical memory processing.

### Predictive Coding and Self-awareness

A potential approach to understand self-awareness across levels of complexity is predictive coding (PC; [56]). Although empirical support for this notion is still emerging [57•], PC links different perspectives on brain function, ranging from the nineteenth-century pioneers [58] to current theoretical approaches [59]. In brief, this framework suggests that, in the absence of complete information about the world and faced with unpredictable conditions, the brain works as an inferential machine [59]. The brain generates predictions (expectancies or ‘beliefs’) that model the likely causes of incoming sensory information, to manage and extract knowledge from the wealth of these afferent signals. The PC framework proposes that the brain generally seeks to minimise mismatch (prediction error, i.e. sensory surprise) between ‘descending’ predictions and ‘ascending’ afferent sensory information. This can be achieved by adjusting the predictions, i.e. by learning (in order to change the beliefs or better trust the prediction through ‘precision weighting’). Alternatively, one can change the incoming sensory information by acting on the world. Here, predictions/beliefs equate to action commands, sequences of which are termed policies. Both learning and ‘active inference’ increase knowledge about the sensory data.

PC accounts for both top-down and bottom-up influences in neural processing. This approach has already been applied in relation to self-awareness, for example in the context of agency [60], interoception [18••], and their potential interaction [61, 62••]. For example, Quadt and colleagues [18••] propose that descending higher-order predictions about inner bodily states, generated cortically, are compared to ascending visceral afferents. Mismatch is used both to refine interoceptive predictions and drive efferent autonomic ‘actions’, to reduce future predictive errors. Representation of self through interoceptive PC is hierarchical; the higher-order integrative representation (of desired internal bodily state based on previous experience) evokes interoceptive policies that drive top-down descending predictions that yoke lower-order homeostatic reflexes and that are constantly compared with bottom-up bodily data.

A related mechanism (plausibly a direct extension of interoceptive PC with active inference, encompassing valenced motivational outcomes) is proposed to support higher-order emotional processing. Again, these principles may operate across varying levels of complexity of self-awareness. The theory of constructed emotion suggests there is an emotional paradox, with nonspecific autonomic activation triggering states that are perceived as a discrete emotion through higher-level appraisal of the (often external) context [63]. Within a PC perspective, a constructed emotion concept is thus a set of predictions about incoming sensory data (including change in interoceptive arousal) [63]. These predictions are again based on previous experience; emotional concepts categorise data patterns to generate discrete emotional experiences, are modified by incoming information, and support allostatic changes in physiology and behaviour, with longer-term adaptive value. In this model, emotional regulation controls the selection of regulatory actions through predictions that re-categorise both sensory data and emotional concepts, [63].

These theoretical formulations propose that top-down processing (i.e. predicting incoming information) is crucial to develop both a sense of interoceptive agency (core biological selfhood) and emotional processing/regulation (self as an emotional agent). Moreover, the same notion likely represents a wider feature of self-awareness: From this perspective, self-awareness can be thought of as the result of predictions dealing with incoming sensory data across different modalities. Top-down expectations, constructed upon previous knowledge and recurrent patterns of interaction with the world, shape and give coherence to bodily information, fostering the emergence of a broader sense of self-awareness. In situations in which predictions broadly match incoming signals, the regular feeling of self-awareness is experienced, whereas mismatches may lead to either adjustment of predictions and behaviour or, in the case of specific neurocognitive symptoms, impaired self-awareness, or delusional explanations for aberrant experiences.

Such a self-model, built through dynamic cross-modal multisensory integration of information, has a high degree of redundancy and, hence, is relatively immune to damage to underlying distributed neural substrates. However, certain sources of sensory data likely have a privileged position in the emergence of self-awareness. For instance, interoceptive imperatives (encoding physiological states necessary for survival) underpin emotional processes [64]. In turn, these contribute to a sense of self-presence and internal agency [61]. Similarly, given the embodied nature of our cognitive capacities, afferent information concerning action and goal-directed behaviour in the external environment may be particularly relevant for the emergence of a sense of self. This places continuous dynamic interoceptive bodily information at the core of a nested-hierarchy for self-representation [4, 5] upon which is built elaborated levels of self-processing and associated self-awareness.

This conceptual model is particularly relevant for the understanding of self-awareness in dementia, considering the profile of global cognitive impairment found in this condition, which may compromise top-down inferential processing of sensory information, and impact the discriminative quality of incoming sensory signals. However, before applying this framework to understand self-awareness in dementia, we review the expression of different facets of self-awareness in the context of neurodegenerative conditions.

## Self-awareness in Dementia

Alzheimer’s disease (AD) is the most common form of neurodegenerative dementia, providing both a strong motivation and a model clinical condition for understanding self-awareness in dementia. AD is a well-characterised neuropsychiatric condition that presents with cognitive, behavioural, and functional deficits that progress in severity with disease duration. The core neuropathological features of AD include widespread neurodegeneration encompassing cortical regions and brain-wise networks that are implicated in supporting aspects of self-awareness. Clinically, people with AD may experience changes to multiple facets of self-awareness. The most well-characterised areas of self-awareness affected by AD are higher levels of autobiographical memory and metacognition.

### Autobiographical Memory

Autobiographical self-awareness relies on both episodic and semantic memory, with the awareness of self that accompanies episodic memory referred to as autonoetic consciousness. This higher-level autobiographical awareness becomes impaired in mild/moderate AD; people with AD have a reduced subjective feeling of re-experiencing the past, with diminished self-imagery and less emotional salience when recalling autobiographical memories [65, 66, 67••]. However, in mild/moderate AD, although there may also be some impaired retrieval of semantic information, there is typically a persistence of semantic knowledge of the self (e.g. elements of personal history) that maintains a preserved narrative sense of self [67••, 68]. This is not always the case for other subtypes of dementia; despite memory impairment in semantic dementia (SD), patients retain feelings of identity for past and present, but not for future selves [69]. Autobiographical memory in behavioural-variant frontotemporal dementia (bvFTD) has been less frequently explored, but results suggest impairments in self-related recollections irrespective of the time period. This may arise from specific compromising of retrieval strategies due to executive deficits [70]. More speculatively, selective depletion of Von Economo neurons in bvFTD may disrupt core interoceptive aspects of self-representation [28].

The narrative sense of self in AD may be associated with reduced awareness of recent changes in cognitive function and behaviour, due to an impaired ability to update and accurately monitor new self-information [71•]. This may give rise to a lack of awareness of new cognitive deficits, or anosognosia, which is a common feature of AD [72]. Anosognosia in AD is associated with neurodegenerative decreases in the structural and functional integrity of distributed brain regions, including frontal cortices, medial temporal lobe, anterior and posterior cingulate cortex, and insula [73••]. Anosognosia in dementia, reflecting limited awareness of a loss in cognitive ability, has also been explored through the concept of metacognition.

### Metacognition

Metacognition, when applied to autobiographical information, can be investigated at a lower, less global level than in overall judgements of cognitive function, by assessing how well an individual can judge their performance accuracy during episodic or semantic memory tasks. In studies of people with early AD, this online monitoring of cognitive performance yields some conflicting results: Metacognitive self-awareness of episodic and semantic memory performance is reported as remaining intact in some [74, 75], but not all, studies where impaired metacognition is expressed as both over- and underestimation of performance [76, 77]. Interestingly, even in cases of limited awareness of performance, PwD can still respond emotionally to tasks [51], persist more during periods of success than periods of failure [78], and allocate study time proportionately to the difficulty of self-recollection [79]. These observations reveal the presence of ‘implicit awareness’ of self [10].

These conflicting results likely reflect how metacognition is defined and assessed, and also to the individual variation of metacognitive self-awareness in people with early AD. This would be underpinned by different profiles of cognitive impairment and underlying neuropathology. Correspondingly, impaired metacognition is particularly associated with localised structural and functional change involving frontal lobes, posterior cingulate cortex [80], and insula [81]. Given the involvement of frontal lobes in metacognition, unsurprisingly comparisons of people with AD and those with frontotemporal dementia (FTD) reveal that the latter group has more extensive metacognitive impairments, especially in bvFTD [82, 83].

### Emotion Regulation

The self-awareness of one’s own emotional state that is necessary for emotion regulation is also affected by dementia. The capacity for emotion regulation has been associated with components of executive function; for example, verbal fluency performance predicts the ability of PwD to regulate a range of emotional responses [84]. However, one study of people with moderate AD found no differences between AD and control groups on either self-reported subjective experience of emotion, nor the ability to inhibit emotion expressive behaviour, although behavioural amplification of emotion was affected [85]. People with AD also show heightened emotional contagion, which is the automatic echoing of the emotional states of others [86]. Affective lability of this sort is also observed following frontal lobe lesions and other conditions compromising response inhibition and impairing emotional regulation. Of note, in a recent study people with AD or FTD were shown films designed to elicit disgust and asked to watch or suppress their emotional response. FTD patients were more impaired in emotion suppression than people with AD or controls. Neuroimaging analysis of brain structure revealed that insula volume predicted the capacity for effective emotion regulation [87].

### Body Ownership and Agency

Few studies have examined evidence of deficits in awareness of body ownership and/or proprioception in people with AD. However, people with dementia appear to show a preserved ability to identify their own body and express agency, which persists even into advanced severe stages of the disease. In one study, all people with moderate AD and 25% of people with severe AD were able to identify parts of their own bodies (e.g. able to indicate their elbow when asked) [88], and people with severe AD are able to use correct personal pronouns (e.g., ‘I’, ‘me’) with reference to themselves [e.g. 89] and to express self-related views [90•]. Relatedly, although studies suggest impairment of mirror self-recognition may occur in severe dementia (e.g. [91]); for example, with PwD seeing themselves in the mirror and thinking there is an intruder, behaviours such as grooming have been cited as evidence of covert self-identification [92].

Additional evidence for the preservation of agency in dementia comes from studies employing a phenomenological perspective, which can be particularly relevant given the constraints to measure agency through cognition in this condition. For instance, an agency in PwD may be observed through the analysis of bodily information, actions, and goal-directed behaviours, beyond standardised cognitive paradigms [93]. Within this framework, people with mild to moderate dementia may present difficulties in decision-making capacity but can demonstrate a sense of agency through behavioral and emotional responses, including emotional reflexivity through expressed feelings and desires, despite limited verbal ability [94].

### Interoception

There is some evidence that interoception may be impaired in AD [95, 96]. In a study using a heartbeat detection paradigm, people with AD demonstrated impairment in levels of interoceptive accuracy and interoceptive awareness, typically overestimating their performance. The study also included a group of people with FTD and frontal stroke with deficits in interoception associated with volume loss in the anterior insula and fronto-temporal lobes [97]. Neurodegeneration of the posterior insula has also been implicated in the reduced interoceptive experience of temperature and pain in people with FTD [97].

### Summary of Findings

There is some evidence that, despite higher-level impairment in autobiographical awareness, lower-level aspects of self-awareness, including indices of body ownership and agency, persist in AD enabling an ongoing persevered global sense of self-awareness (summarised in Table 2). Clinical evidence from people with AD nevertheless illustrates the multi-level and multi-faceted nature of self-awareness. The heterogeneity of self-awareness in AD, particularly at higher-order levels, is further supported by studies that assessed distinct targets of self-awareness [98, 99]. One study quantified awareness, through the discrepancy between carers and PwD evaluation, of cognitive dysfunction, illness severity, emotional state, daily functioning, and social relationships [99]. The discrepancy scores differed between domains, interpreted as reflecting the heterogeneity of self-awareness in people with mild and moderate AD. Moreover, although levels of overall awareness, awareness of cognitive problems, and impaired functioning were worse in moderate compared to mild AD, there was no significant difference between moderate and mild AD groups in awareness of emotional state [99]. These data provide evidence for domain-selective deficits in self-awareness in PwD, which in turn may relate to the patterned distribution of neurodegenerative processes and impact on the different demands of self-perceptions and judgements reflecting self-awareness in distinct domains [6, 99].

**Table 2:** Self-awareness in dementia

Self-awareness processes	Evidence in PwD	Clinical expression
Interoception	Impairments in interoceptive accuracy and awareness	Difficulties in estimating internal bodily states; overestimation of the interoceptive capacity
Proprioception and body ownership	Identification of own body and covert mirror self-recognition even in severe dementia	Use of personal pronouns, expression of self-related views; grooming in front of mirrors when prompted with cues
Agency	Impairments in the cognitive agency, but preservation through behavioural and emotional responses	Expression of feelings and desires even in cases of compromised language ability
Metacognition	Discrepant findings, but impairments especially in cases of damage to frontal lobes	Uncertainty when describing personal ability; implicit adjustment to task difficulty
Emotional regulation	Impairments in suppression (FTD) or amplification of emotions (AD); heightened emotional contagion	Difficulties controlling emotions; agitation and anger
Autobiographical memory	Impairments in mild to moderate AD, in particular of episodic components; reverse memory gradient in semantic dementia; strategic retrieval deficits in bv FTD	Persistent self-narratives, especially about the past; difficulties in updating self-information

## Towards an Integrative Framework of Self-awareness in Dementia

An important feature of selfhood in dementia is its typical persistence at a core level, even in severe stages of neurodegeneration. Few attempts have been made to understand this phenomenon from a neurobiological perspective, but recently, the notion of an emergent self is suggested to account for this [100••]. According to this perspective, the feeling of self is but the combination of a set of processes, with experiences from different sources, such as interoception, agency, autobiographical memory, and metacognition, leading to a higher-order property, including a unified phenomenological experience. This refers to a fundamental feature of our consciousness, namely its binding ability, or the sense of perceiving an experience as a unified whole instead of a combination of fragmentary elements. In dementia, this sense of selfhood may emerge from the combination of bodily, agentic, implicit, and mnemonic information, as well as recognising the important contributions of a surrogate (others) and extended (the environment) sources [100••]. The variety of these sources may explain the resilience of selfhood even towards later stages of dementia, highlighting that some of these processes may compensate for the impairment of others. At the neural level, a self-model, arising initially from interoceptive control processes and subsequently elaborated through dynamic cross-modal multisensory integration of diverse information across distributed neural substrates, may retain a high degree of redundancy and remain relatively immune to neurodegenerative damage.

Combining the notion of diverse self-processes that may lead to a solid sense of self in dementia, with a PC framework, provides the ground for the discussion of an integrative model of self-awareness in dementia. This model would embody notions of nested hierarchy within self-processes [4, 5], but and benefit from also from a finer empirical appreciation of how top-down and bottom-up self-referential processing may be affected by dementia.

Considering top-down influences, it is noted that PwD has stable but outdated personal information about themselves, with this having a likely impact on the estimation of self-ability. This is termed as a metaphor, a ‘petrified self’ [71•], and refers to the profile of mnemonic impairment caused by hippocampal damage: anterograde memory deficits coupled with retrograde memory difficulties with a gentle temporal gradient. Within a PC perspective, this may be linked to predictions, which are stable but, nevertheless, are not updated efficiently with incoming information.

In terms of future research, our formulation provides a blueprint for empirical studies aiming to explore self-awareness in dementia. Considering top-down mechanisms, investigating how the profile of cognitive impairment in different stages of dementia impact the formation of predictions may illuminate distortions of self-awareness. A key prediction, consistent with current findings, is that higher-order features of self-awareness would be more impaired than lower-order processes. This may also help to explain the presence of psychological and behavioural symptoms of dementia, including psychotic symptoms (e.g. delusions, misidentification), which may emerge as an attempt to make sense of mismatches between predictions and sensory data.

In addition, exploring the level of preservation of different sensory signals can crucially help understand impaired self-awareness in dementia. In particular, studies with PwD will benefit from detailed characterization of low-level processing of interoceptive, body ownership, and agency sensory data, given the potential resilience of these processes in later stages of the condition and also how these may feed into higher-order self-awareness. For example, different aetiologies of dementia may be linked to diverse alterations of interoception, which may underlie changes in other forms of self-awareness, such as emotional regulation. The extent to which this may alter selfhood in dementia is especially relevant from a clinical perspective.

## Conclusion

As indicated, self-awareness can be thought of as a constellation of different processes, which interact and are typically integrated to give rise to a unitary experience. These processes happen within a continuum of complexity, involving lower- and higher-order information. Evidence from PwD reinforce this notion, by highlighting the presence of a mixed profile of impairment and preservation, depending on the self-awareness process investigated and its level, moderated by clinical factors including the pathoetiological nature of dementia. The notion of an emergent self [100••] may help to explain, from a neurobiological perspective, how interaction between self-awareness processes may compensate for impaired self-awareness abilities and maintain a resilient sense of selfhood in PwD.

Exploring the diversity of self-awareness processes across dementia severity levels may help to determine the time course of alterations, with initial higher-order impairment linked to cortical damage and, later on, alterations associated with bottom-up processing. Clinically, such a research programme would have as its ultimate goal the development of care practices that acknowledge the extent of preservation of self-awareness in dementia and capitalise on preserved abilities to foster and maintain personhood as the condition progresses.
